# Early onset lung cancer, cigarette smoking and the SNP309 of the murine double minute-2 (*MDM2*) gene

**DOI:** 10.1186/1471-2407-8-113

**Published:** 2008-04-23

**Authors:** Kirstin Mittelstrass, Wiebke Sauter, Albert Rosenberger, Thomas Illig, Maria Timofeeva, Norman Klopp, Hendrik Dienemann, Eckart Meese, Gerhard Sybrecht, Gabi Woelke, Mathias Cebulla, Maria Degen, Harald Morr, Peter Drings, Andreas Groeschel, Karsten Grosse Kreymborg, Karl Haeußinger, Gerd Hoeffken, Christine Schmidt, Bettina Jilge, Wilhelm Schmidt, You-Dschun Ko, Dagmar Taeuscher, Jenny Chang-Claude, Heinz-Erich Wichmann, Heike Bickeboeller, Angela Risch

**Affiliations:** 1 Helmholtz Center Munich, German Research Center for Environmental Health, Institute of Epidemiology, Munich/Neuherberg, Germany; 2 Department of Genetic Epidemiology, Georg-August University of Göttingen, Medical School, Göttingen, Germany; 3 German Cancer Research Centre (DKFZ), Heidelberg, Germany; 4 Thoraxklinik Heidelberg GmbH, Heidelberg, Germany; 5 Institute of Human Genetics, University of the Saarland, Germany; 6 Institute of Internal Medicine V, Medical School, University of the Saarland, Germany; 7 Municipal Hospital „Sankt Georg", Leipzig, Germany; 8 Clinic of Pneumology, Waldhof Elgershausen, Greifenstein, Germany; 9 Department Internal Medicine-Oncology, Thorax-Clinic Heidelberg GmbH, Heidelberg, Germany; 10 Centre for Internal Medicine, University Hospital Leipzig, Germany; 11 Clinic of Pneumology München-Gauting, Munich, Germany; 12 Clinic Coswig GmbH, Division Internal Medicine, Coswig, Germany; 13 Chemnitz Hospital, Clinic for Internal Medicine, Chemnitz, Germany; 14 Department of Oncology, University Hospital of Bonn, Germany; 15 Wald-Hospital Gera GmbH, II. Medical Clinic, Gera, Germany; 16 Ludwig-Maximilians-University of Munich, Institute of Medical Data Management, Biometrics and Epidemiology, Germany

## Abstract

The polymorphism SNP309 (rs2279744) in the promoter region of the *MDM2 *gene has been shown to alter protein expression and may play a role in the susceptibility to lung cancer. The MDM2 protein is a key inhibitor of p53 and several mechanisms of MDM2/p53 interactions are presently known: modulating DNA-repair, cell-cycle control, cell growth and apoptosis.

We used 635 Caucasian patients diagnosed with lung cancer before 51 years of age and 1300 healthy gender and age frequency matched population Caucasian controls to investigate the association between the *MDM2 *SNP309 and the risk of developing early onset lung cancer. Conditional logistic models were applied to assess the genotype-phenotype association, adjusted for smoking.

Compared to the GG genotype, the adjusted ORs for the TG and TT genotype were 0.9 (95% CI: 0.7–1.5) and 1.0 (95% CI: 0.7–1.5), respectively. Also no association was found for histological subtypes of lung cancer. The strength of this study is that within young cases the genetic component to develop lung cancer may be greater. Our results indicate that the *MDM2 *SNP309 is not significantly associated with lung carcinogenesis but point towards gender-specific differences.

## Background

Human cells have developed a complex system to protect themselves from genotoxic damage where the *p53 *tumor suppressor gene plays an important role in protecting against such insults by serving as an integrator of the signals produced by DNA damage. The MDM2 protein inhibits p53 transactivation activity and promotes its export from the nucleus by binding to the transcriptional activation domain of p53 [1] and acts as an ubiquitin ligase upon p53 pushing fast degradation of the suppressor [2,3].

Recently, a SNP (SNP309, G2580T) at the intronic p53-response promoter of *MDM2 *was identified and associated with altered Sp1 binding affinity and expression levels of MDM2 RNA and protein [4]. Contradictory results were reported regarding the *MDM2 *SNP309 association with lung cancer [5-8]. In addition the polymorphism was correlated with a decreased age at the time of lung cancer diagnosis in Li-Fraumeni syndrome and sporadic sarcoma patients [4]. Our study is the first report on a large German case-control study of young lung cancer patients (age of onset < 51 years) investigating the *MDM2 *SNP 309.

## Methods

### Study population

The study population consists of 635 lung cancer patients with an onset of disease ≤ 50 years of age and 1300 healthy population controls, all Caucasians. Controls were recruited from the KORA S3 + S4 (Cooperative Health Research in the Augsburg Region) surveys, which are large population-based consecutive cross-sectional studies [9,10] and matched by gender and 3-years age groups to cases in a 1:2 matching design. In order to increase the sample size of early onset lung cancer patients we included cases from two existing studies. 472 cases were from the LUCY (LUng Cancer in the Young) study. Additional 163 cases were derived from the hospital-based Heidelberg Lung Cancer (HLC) study from the Thoraxklinik Heidelberg [11].

The **LUCY-study **is a multicenter study with 31 participating clinics all over Germany. Only newly diagnosed patients with histological or cytological confirmed primary lung cancer entered the study. Detailed epidemiologic data on family history, tobacco and smoking exposure, education and occupational exposure have been collected and blood samples were taken.

The **HLC-study **is an ongoing hospital based case-control study. The DKFZ has recruited over 1000 LC cases at and in collaboration with the Thoraxklinik Heidelberg. 163 of these LC cases with onset of disease under age 51, recruited between 01/1997 and 12/2003 were included in the analysis. Data on occupational exposure, tobacco smoking and educational status were assessed by a self-administered questionnaire.

The **KORA-study **is a population based epidemiological survey of persons living in or near the city of Augsburg, Southern Germany conducted since 1984. No major population stratification between KORA (Southwest Germany) and two other cohorts from Northern Germany could be detected in an intensive study using a genomic control approach [12].

Informed consent was obtained from all study participants and the studies were approved by the ethical committee of the Bayerische Landesärztekammer, the corresponding local ethics committees of the participating clinics and the ethical committee of the University of Heidelberg.

### Genotyping

The detection of the *MDM2 *gene polymorphism (rs2279744; G > T; NM_002392.2) was based upon analysis of primer extension products generated from previously amplified genomic DNA using a chip-based MALDI-TOF (matrix-assisted laser desorption/ionization time-of flight) mass spectrometry platform (SEQUENOM, Inc., San Diego, CA) as described in Weidinger et al. (2004) [13]. Genotyping was performed by laboratory personnel blinded to case-control status. Standard genotyping quality control included 10% duplicate samples, negative and positive samples and checking for Hardy-Weinberg equilibrium (HWE).

### Statistical analysis

Hardy Weinberg Equilibrium (HWE) was tested using a log-likelihood test. Conditional logistic regression models were applied to test for a genotype-phenotype association and gene-smoking interaction, with conditioning on the matched variables age groups (≤ 40 years, 41–45 years, 46–50 years) and gender. The amount of smoking exposure (s. table 1 for definition) was considered as a covariate to adjust for a smoking-related increase in lung cancer risk. The overall level of significance was set to 5%. We investigated differences between multiple characteristics (age of onset, gender, LC subtype and *MDM2 *SNP390 genotype) of both cases-samples by calculating a propensity score, which is defined as the conditional probability of assignment to a particular case-sample given a vector of observed covariates [14]. We then calculated kappa as measurement of agreement between true and the assigned membership to a case-sample and performed a McNemar's test for agreement between these.

**Table 1:** Characteristics of lung cancer cases and healthy controls

**Category**	**Group**	**Cases (n = 635)**		**Controls**
		
		**LUCY-study** **(n = 472)**	**HLC-study** **(n = 163)**	**KORA-study** **(n = 1300)**
gender	men	66% (310)	59% (96)	63% (819)
	women	34% (162)	41% (67)	37% (481)

age at diagnosis (mean ± SD)	men	45.4 ± 4.1	45.4 ± 3.7	45.2 ± 4.3
	women	44.5 ± 4.6	45.1 ± 4.6	44.7 ± 4.7

age at diagnosis (men)	≤ 40 years	13% (40)	11% (11)	14% (116)
	41–45 years	32% (99)	33% (32)	31% (251)
	46–50 years	55% (171)	55% (53)	55% (451)

age at diagnosis (women)	≤ 40 years	17% (28)	16% (11)	17% (83)
	41–45 years	31% (51)	25% (17)	30% (144)
	46–50 years	51% (83)	58% (39)	53% (255)

LC subtypes (men)	NSCLC-Adenocarcinoma	26% (81)	35% (34)	
	other NSCLC	40% (125)	43% (41)	
	SCLC	26% (81)	20% (19)	
	other types	7% (22)	2% (2)	

LC subtypes (women)	NSCLC-Adenocarcinoma	44% (71)	54% (36)	
	other NSCLC	22% (36)	24% (16)	
	SCLC	25% (41)	16% (11)	
	other types	9% (14)	6% (4)	

smoking exposure (packyears)	minimally exposed (0–10)	12% (57)	13% (21)	54% (712)
	lightly exposed (11–20)	17% (81)	8% (13)	14% (178)
	moderately exposed (21–30)	31% (144)	20% (33)	15% (188)
	highly exposed (31++)	39% (186)	52% (85)	15% (192)
	unknown	0.9% (4)	7% (11)	2% (30)

*MDM2 *SNP309 genotypes*	TT	42% (199)	44% (71)	42% (547)
	TG	47% (222)	44% (71)	46% (598)
	GG	10% (49)	13% (21)	12% (149)
	unknown	0.3% (2)	--	0.5% (6)

mean age at diagnosis (95% CI)	TT	45.1 (44.5–45.7)	44.8 (43.7–45.8)	
	TG	45.3 (44.7–45.8)	45.6 (44.7–46.5)	
	GG	44.6 (43.3–45.9)	45.9 (44.2–47.6)	

LUCY-study: Lung Cancer in the Young study; HLC-Study: Heidelberg lung cancer study; Other NSCLC = other non small cell lung cancers (NSCLC excluding adenocarcinoma), SCLC = small cell lung cancer, other types = other kinds of lung cancer

All analyses were performed with SAS 9.1^®^. Power analyses for the single marker association was done according to the method of Slager 2001 [15] and for the single marker interaction tests it was accomplished by using Nquery Advisor 4.0.

## Results

### Population characteristics

There was no statistically significant difference in the distribution of gender (p = 0.13) and age (p = 0.80) between patients and controls. The distribution of the histology subtypes within the cases can be seen in table 1. Although one might see a higher percentage of NSCLC-Adenocarcinoma within HCL-cases, the difference between both case-populations was by far not significant (p = 0.617). In total, there are no major differences between both case-populations. Applying the propensity score approach we found no agreement between the true and the assigned membership to a case-sample (kappa 0.01; 95% CI: -0.1 to 0.1). Thus we have no evidence to assume group differences shown by the multiple characteristics considered (p = 1.00).

### Genotyping

The genotyping success rates were above 99.5%. No discordance between the duplicates was found.

### Associations of the MDM2 SNP309 with lung cancer

No deviations from HWE were found in the genotype distribution of the *MDM2 *polymorphism among controls (p = 0.453) or cases (p = 0.474). All but eight samples were successfully genotyped for the *MDM2 *SNP309. The minor allele frequency (MAF) was 0.34 (CI: 0.35–0.33). Lung cancer was not found to be associated with the *MDM2 *SNP309 (p = 0.988). Compared to the GG genotype, the smoking adjusted ORs for the TG and TT genotypes were 1.0 (95% CI: 0.7–1.5) and 1.0 (95% CI: 0.7–1.5), respectively. Both case populations compared separately to the controls showed no significant associations (Table 2). Gender related subgroup analysis yielded no association of the *MDM2 *SNP309 and lung cancer (Table 2, 3, 4). Also no influence in lung cancer risk by the *MDM2 *SNP309 was observed for the diverse histological subtypes of lung cancer (Table 3, 4, 5).

**Table 2:** Association of *MDM2 *SNP309 with lung cancer within both case-populations

**genotype**	**OR**	**95% CI**	**p-value**
**LUCY **cases (n = 470) vs. KORA controls (n = 815)			
TG vs. GG	1.1^§^	0.7–1.6	0.794
TT vs. GG	1.0^§^	0.7–1.6	0.841
**men**: LUCY cases (n = 309) vs. KORA controls (n = 815)			
TG vs. GG	1.1^§§^	0.7–1.8	0.706
TT vs. GG	1.1^§§^	0.7–1.8	0.751
**women**: LUCY cases (n = 161) vs. KORA controls (n = 815)			
TG vs. GG	1.0^§§^	0.5–2.1	0.937
TT vs. GG	1.0^§§^	0.5–2.1	0.966
gender difference in genotypic ORs			0.778

**HLC **cases (n = 163) vs. KORA controls (n = 479)			
TG vs. GG	0.9^§^	0.5–1.7	0.761
TT vs. GG	1.0^§^	0.5–1.7	0.906
**men**: HLC cases (n = 96) vs. KORA controls (n = 479)			
TG vs. GG	1.2^§§^	0.5–3.0	0.648
TT vs. GG	1.6^§§^	0.7–3.7	0.314
**women**: HLC cases (n = 67) vs. KORA controls (n = 479)			
TG vs. GG	0.7^§§^	0.3–1.6	0.372
TT vs. GG	0.5^§§^	0.2–1.3	0.159
gender difference in genotypic ORs			0.083

**HLC **cases vs. **LUCY **cases (distribution of TT/TG/GG)			
all			^$^0.773
men			^$^0.383
women			^$^0.100

^§ ^conditional to age/gender-strata (6 groups) and adjusted for smoking exposure^§§ ^conditional to age-strata (3 groups) and adjusted for smoking exposure and gender^$^. Fishers Exact Test

**Table 3:** Association of *MDM2 *SNP309 with lung cancer

**gender**	**Genotype**	**OR**	**95% CI**	**p-value**
men*	TG vs. GG	1.1	0.7 – 2.0	0.630
men*	TT vs. GG	1.2	0.7 – 2.2	0.504
women*	TG vs. GG	0.9	0.5 – 1.6	0.723
women*	TT vs. GG	0.8	0.5 – 1.3	0.565

**histology**				

NSCLC-Adenocarcinoma**	TG vs. GG	1.0	0.6 – 1.7	0.991
NSCLC-Adenocarcinoma**	TT vs. GG	1.0	0.6 – 1.8	0.890
other NSCLC**	TG vs. GG	1.0	0.6 – 1.8	0.873
other NSCLC**	TT vs. GG	0.9	0.5 – 1.6	0.751
SCLC**	TG vs. GG	1.0	0.5 – 1.9	0.980
SCLC**	TT vs. GG	1.2	0.6 – 2.1	0.637

**smoking exposure (packyears)**				

lightly exposed (11–20 PY)***	TT vs. GG	1.0	0.6 – 1.8	0.928
lightly exposed (11–20 PY)***	TG vs. GG	1.0	0.5 – 1.7	0.857
moderately exposed (21–30 PY)***	TT vs. GG	1.0	0.6 – 1.7	0.960
moderately exposed (21–30 PY)***	TG vs. GG	0.9	0.6 – 1.6	0.822
highly exposed (31++ PY)***	TT vs. GG	0.9	0.5 – 1.4	0.595
highly exposed (31++ PY)***	TG vs. GG	0.8	0.5 – 1.3	0.369

*conditional to age-strata (3 groups: ≤ 40 years, 41–45 years, 46–50 years), adjusted for smoking exposure; **conditional to age/gender-strata (6 groups) and adjusted for smoking exposure; ***conditional to age/gender-strata (6 groups)

**Table 4:** Association of *MDM2 *SNP309 with lung cancer within NSCLC cases

**genotype**	**OR**	**95% CI**	**p-value**
**all**: NSCLC cases (n = 434) vs. controls (n = 1295)			
TG vs. GG	1.1^§^	0.7–1.6	0.828
TT vs. GG	1.0^§^	0.7–1.5	0.841
**men**: NSCLC cases (n = 280) vs. controls (n = 815)			
TG vs. GG	1.2^§§^	0.7–2.9	0.422
TT vs. GG	1.2^§§^	0.7–2.9	0.515
**women**: NSCLC cases (n = 154) vs. controls (n = 479)			
TG vs. GG	0.8^§§^	0.4–1.6	0.471
TT vs. GG	0.7^§§^	0.4–4.0	0.323
gender difference in genotypic ORs			0.237

^§ ^conditional to age/gender-strata (6 groups) and adjusted for smoking exposure^§§ ^conditional to age-strata (3 groups) and adjusted for smoking exposure and gender

**Table 5:** *MDM2 *SNP309 genotypes

***Group***	***MDM2 SNP309 genotypes***		**Cases (n = 635)**		***Controls***
			
			**LUCY-study** **(n = 472)**	**HLC-study** **(n = 163)**	**KORA-study** **(n = 1300)**
gender	men	TT	128 (41%)	47 (49%)	350 (43%)
		TG	148 (48%)	42 (44%)	366 (45%)
		GG	33 (11%)	7 (7%)	99 (12%)
		unknown	1 (0%)		3 (0%)
	women	TT	71 (44%)	24 (36%)	197 (41%)
		TG	74 (46%)	29 (43%)	232 (48%)
		GG	16 (10%)	14 (21%)	50 (10%)
		unknown	1 (0%)		3 (0%)

age at diagnosis	≤ 40 years	TT	30 (44%)	11 (50%)	85 (43%)
		TG	30 (44%)	8 (36%)	91 (46%)
		GG	6 (9%)	3 (14%)	21 (11%)
		unknown	2 (3%)		2 (1%)
	41–45 years	TT	58 (39%)	21 (43%)	172 (44%)
		TG	73 (49%)	23 (47%)	185 (47%)
		GG	19 (13%)	5 (10%)	37 (9%)
		unknown			1 (0%)
	46–50 years	TT	111 (44%)	39 (42%)	290 (41%)
		TG	119 (47%)	40 (43%)	322 (46%)
		GG	24 (9%)	13 (14%)	91 (13%)
		unknown			3 (0%)

smoking exposure level (SEL)	minimally exposed	TT	28 (49%)	7 (33%)	297 (42%)
	(0–10 PY)	TG	19 (33%)	12 (57%)	332 (47%)
		GG	9 (16%)	2 (10%)	81 (11%)
		unknown	1 (2%)		2 (0%)
	lightly exposed (> 10–20 PY)	TT	29 (36%)	7 (54%)	72 (40%)
		TG	42 (52%)	5 (38%)	81 (46%)
		GG	9 (11%)	1 (8%)	24 (13%)
		unknown	1 (1%)		1 (1%)
	moderately exposed (> 20–30 PY)	TT	65 (45%)	13 (39%)	81 (44%)
		TG	70 (49%)	15 (45%)	86 (46%)
		GG	9 (6%)	5 (15%)	19 (10%)
		unknown			
	highly exposed	TT	76 (41%)	41 (48%)	84 (44%)
	(> 30++ PY)	TG	89 (48%)	33 (39%)	87 (45%)
		GG	21 (11%)	11 (13%)	21 (11%)
		unknown			

LC subtypes	NSCLC- Adenocarcinoma	TT	63 (41%)	35 (50%)	
		TG	75 (49%)	23 (33%)	
		GG	13 (9%)	12 (17%)	
		unknown	1 (1%)		
	other NSCLC	TT	68 (43%)	17 (30%)	
		TG	73 (46%)	34 (60%)	
		GG	19 (12%)	6 (11%)	
	SCLC	TT	55 (45%)	15 (50%)	
		TG	55 (45%)	12 (40%)	
		GG	13 (11%)	3 (10%)	
	other types	TT	13 (27%)	4 (40%)	
		TG	19 (39%)	2 (20%)	
		GG	4 (8%)		
		unknown	13 (27%)	4 (40%)	

Furthermore it was investigated whether an interaction existed between the MDM2 SNP309 and the smoking status. We did not find an association between the MDM2 SNP309 and lung cancer for any of the defined smoking exposure level groups (Table 3, 5).

Even though no significant difference between both case-populations could be stated statistically, we observed twice as many female GG-carriers among HLC-cases (21%) than in the control- or LUCY population (10%) (Table 5). We also observed an enrichment of NSCLC- and NSCLC adenocarcinoma-cases in the HLC-sample (78%) compared to the LUCY-sample (66%). Lind et al. (2006) [6] recently reported a gender specific risk disposing effect of the T-allele of *MDM2 *SNP309 for NSCLC cases. We therefore additionally performed a subgroup analysis restricted to NSCLC-cases and tested for gender differences. Compared to the GG genotype, the smoking adjusted ORs for the TG and TT genotypes for female NSCLC-cases were 0.8 (95% CI: 0.4–1.6) and 0.71 (95% CI: 0.4–4.0), respectively. For men the ORs were 1.2 (95% CI: 0.7–2.8) and 1.2 (95% CI: 0.7–2.9), respectively (Table 4). Although a possible NSCLC risk modification of *MDM2 SNP309 *seems to be reverse between men and women, we failed to achieve significance for this gender difference (p = 0.237). We also tested a gender specific genetic association within both case-samples separately. Neither within all HLC-cases (p = 0.083) nor within all LUCY-cases (p = 0.778) were significant gender differences found (Table 2).

*MDM2 *SNP309 was recently reported to be associated with an earlier onset of disease [4]. The average age at the time of diagnosis in our cases overall was not significantly different (p = 0.923) between the variant genotypes (Table 1) and can be assumed as homogeneous between both case-samples (p = 0.7157). The mean age of diagnosis of men was 45.2 (median = 46.0 years, range 26.0 – 50.0 years) and of women 44.7 years (median = 46.0 years, range 24.0 – 50.0 years). Women, however, with the *MDM2 *SNP309 were diagnosed at a younger age (8 months) compared to males, but this was not statistically significant (p = 0.059, 95% CI: 0 to 16 months). Within the LUCY-case population this gender difference was 10 months (95% CI: 0.3 to 20 months, p = 0.044), and only 6 months (95% CI: -10 to 22 months, p = 0.496) in the much smaller HLC-case population.

## Discussion

Reports have shown an increased risk of lung cancer for the G allele of the *MDM2 *SNP309 in Korean and Chinese populations [7,16,17]. In contrast, in our study of 635 Caucasian lung cancer patients with age of onset < 51 years and 1300 controls the *MDM2 *SNP309 was not associated with lung cancer. We did not observe a significant overall association either with the G allele or with the T allele as reported by Li et al. (2006) where they showed an increased lung cancer risk with the T/T genotype in a non-Hispanic white population [18]. We had a 90% power (alpha = 0.05, two sided test) to detect a minimum overall OR of 1.4 for the *MDM2 *SNP309 T allele. Thus our study had sufficient power to detect an OR of 1.6 (95% CI: 1.1–2.5) and 1.8 (95% CI: 1.5–2.3) reported in other studies [6,7]. The estimated allele frequency (T: 0.66, G: 0.34) agrees well with further reported allele frequencies in Caucasian populations [6,8]. Differences between the two case populations were statistically not palpable (Table 1, 2, 3, 4, 5) but the HLC-sample was enriched with NSCLC-cases. According to Steffens et al. 2006 [12] we can assume the German population to be nearly free of population stratification, which may lead to false positive or false negative association findings in genetic case control studies. Differences in ethnicity between our population and those reported by Zhang et al. 2006 [7], Park et al. 2006 [16] and Jun et al. 2007 [19] may provide a partial explanation for the lack of association in our study. On the other hand Hu et al. 2006 [5] also did not observe an association between the *MDM2 *SNP309 and lung cancer in a Chinese population. Our results were similar to the report of Pine et al. (2006) [8]. They also did not find any overall association between the *MDM2 *SNP309 and lung cancer in a Caucasian (USA) population. Similarly, we did not observe an interaction between the *MDM2 *SNP309 and smoking. A combined analysis of the available genotype data of the *MDM2 *SNP309 showed an increased risk of lung cancer for the GG versus the TT genotype (OR: 1.3, 95% CI: 1.1–1.4) [20]. A main difference between the two study populations is the age of cases. Therefore one possible explanation would be, that the *MDM2 *variant is only associated with risk of later onset lung cancer. On the other hand a large study of 1787 Caucasian lung cancer patients could also not confirm the results of this meta-analysis. They did not find any significant association of the *MDM2 *SNP309 and lung cancer [21]. The populations used in the meta-analysis are mostly of Asian origin so possibly pointing to population heterogeneity.

Lind et al. (2006) [6] recently reported a gender specific risk disposing effect of the T allele of *MDM2 *SNP309 for NSCLC cases. They investigated NSCLC-cases and found female carriers of the GG-genotype associated to LC by an OR of 4.1, while male homozygotes showed a non significant OR of 1.3. Our data slightly tends to confirm this finding, but the corresponding OR for NSCLC women is just 1.4 (1/0.71 of Table 4), which is much smaller and not significant (p = 0.323). In our study none of the gender specific ORs nor a test of gender differences yielded significance. But we noticed discrepancies of such a gender specific genetic association between the two case-samples of our investigation. A gender difference was almost significant (p = 0.08) for the much smaller single center case-sample of the HLC-study. There, the OR for female carriers of the GG-Genotype (corresponding to Lind et al. (2006)) was 1.9 (1/0.54 of Table 4) and for male carriers 0.6 (1/1.56 of Table 4). With n = 67 female and n = 96 male cases these subsamples are quite small and the tests for association are underpowered. Thus our investigation provides no significance for such gender specific associations by itself. Comparing our study samples to that of Lind et al. (2006) the major difference is the age of onset of LC. The presented investigation focused on early onset LC cases which are in the mean 20 years younger than those of Lind et al. (2006). As the population by Lind et al. (2006) is of Norwegian origin there might also be different gene-environmental interactions.

*MDM2 *SNP309 was reported by Bond et al. 2004 [4] to be associated with an earlier onset of disease for Li-Fraumeni syndrome and sporadic sarcoma patients. We did not observe a lower age of diagnosis in our study, similar findings are also described by Hu et al. 2006 [5] and Pine et al. 2006 [8]. Our sample size is large enough to detect a shift of two years for the time of diagnosis with 90% power (alpha = 0.05) for heterozygous as well as for homozygous allele carriers. Recently Bond et al. 2006 [22] reviewed the possible interaction between the SNP309 and the oestrogen receptor status in women. Within women of the LUCY case population we could observe an earlier onset of disease of 10 months (95% CI: 0.3 to 20 months, p = 0.044), but only 6 months (95% CI: -10 to 22 months, p = 0.496) in the by far smaller HLC-case population. Recent data suggest that the promoter polymorphism in the *MDM2 *gene may influence the age of cancer onset in a gender specific way [23-25].

## Conclusion

Given that lung cancer is a result of gene-environmental interaction, the genetic component may be a particularly strong risk factor among early onset lung cancer patients. In our sample, after controlling for gender and other characteristics, the overall data suggest that the *MDM2 *SNP309 might not be a sufficient risk factor of lung carcinogenesis not even in young cases where the genetic component might have a larger contribution to the risk of disease. It might modify the time of tumour onset, especially in women, supporting the model, that female-specific hormones, such as oestrogen, could allow the SNP309 to accelerate tumour formation. Our investigation provides no significance for gender specific associations by itself; it points only towards gender specific differences. Therefore further studies have to be conducted to explicitly study these gender-specific effects.

## Abbreviations

CI: confidence interval; HLCS: Heidelberg lung cancer study; KORA: Cooperative Health Research in the Augsburg Region; LC: lung cancer; LUCY: lung cancer in the young; MALDI-TOF: matrix-assisted laser desorption/ionization time-of flight; NSCLC: none small cell lung cancer; OR: odds ratio; PY: packyear; SCLC: small cell lung cancer.

## Competing interests

The authors declare that they have no competing interests.

## Authors' contributions

KM carried out the molecular genetic study and drafted the manuscript. WS, NK carried out the molecular genetic study. AR, JCC performed the statistical analysis. HEW, HB, ARi, TI conceived the study, and participated in its design and coordination. GW proband recruitment and data acquisitions. MT data acquisitions. NK, HD, EM, GS, MC, MD, HM, PD, AG, KG, KH, GH, CS, BJ, WS, YK, DT recruited the lung cancer cases in different hospitals. All authors read and approved the final manuscript.

## Pre-publication history

The pre-publication history for this paper can be accessed here:


